# Efficient Purification of Polyhistidine-Tagged Recombinant Proteins Using Functionalized Corundum Particles

**DOI:** 10.3390/biotech12020031

**Published:** 2023-05-03

**Authors:** Jule L. Völzke, Sarah Smatty, Sarah Döring, Shireen Ewald, Marcus Oelze, Franziska Fratzke, Sabine Flemig, Zoltán Konthur, Michael G. Weller

**Affiliations:** Federal Institute for Materials Research and Testing (BAM), Richard-Willstätter-Strasse 11, 12489 Berlin, Germanyzoltan.konthur@bam.de (Z.K.)

**Keywords:** aluminum oxide, sapphire, ethylenediaminetetraacetic acid, nickel chelate, EDTAD, hexaHis-Tag, His6, 6xHis, His8, bioseparation, IMAC purification, immunocapture, affinity chromatography, carrier, nickel, recombinant protein, *Escherichia coli*, bacterial lysates, cytoplasm

## Abstract

Immobilized metal affinity chromatography (IMAC) is a popular and valuable method for the affinity purification of polyhistidine-tagged recombinant proteins. However, it often shows practical limitations, which might require cumbersome optimizations, additional polishing, and enrichment steps. Here, we present functionalized corundum particles for the efficient, economical, and fast purification of recombinant proteins in a column-free format. The corundum surface is first derivatized with the amino silane APTES, then EDTA dianhydride, and subsequently loaded with nickel ions. The Kaiser test, well known in solid-phase peptide synthesis, was used to monitor amino silanization and the reaction with EDTA dianhydride. In addition, ICP-MS was performed to quantify the metal-binding capacity. His-tagged protein A/G (PAG), mixed with bovine serum albumin (BSA), was used as a test system. The PAG binding capacity was around 3 mg protein per gram of corundum or 2.4 mg per 1 mL of corundum suspension. Cytoplasm obtained from different *E. coli* strains was examined as examples of a complex matrix. The imidazole concentration was varied in the loading and washing buffers. As expected, higher imidazole concentrations during loading are usually beneficial when higher purities are desired. Even when higher sample volumes, such as one liter, were used, recombinant protein down to a concentration of 1 µg/mL could be isolated selectively. Comparing the corundum material with standard Ni–NTA agarose beads indicated higher purities of proteins isolated using corundum. His6-MBP-mSA2, a fusion protein consisting of monomeric streptavidin and maltose-binding protein in the cytoplasm of *E. coli*, was purified successfully. To show that this method is also suitable for mammalian cell culture supernatants, purification of the SARS-CoV-2-S-RBD-His8 expressed in human Expi293F cells was performed. The material cost of the nickel-loaded corundum material (without regeneration) is estimated to be less than 30 cents for 1 g of functionalized support or 10 cents per milligram of isolated protein. Another advantage of the novel system is the corundum particles’ extremely high physical and chemical stability. The new material should be applicable in small laboratories and large-scale industrial applications. In summary, we could show that this new material is an efficient, robust, and cost-effective purification platform for the purification of His-tagged proteins, even in challenging, complex matrices and large sample volumes of low product concentration.

## 1. Introduction

The production of recombinant proteins has undergone major improvements in recent years. Proteins, originating from prokaryotic and eukaryotic expression in cell cultures, can now be produced on a large scale, such as kilograms to tons [1,2]. Mainly human antibodies are manufactured in this way, creating new powerful tools in cancer therapy [3,4], just like proteins for vaccine development [5,6]. Recombinant proteins also play an increasing role in bioanalytical test formats such as ELISA (Enzyme-linked Immunosorbent Assay), and lateral flow assays (LFAs). A main bottleneck for these applications is the production of purified target proteins from adequate expression systems [7]. After successful recombinant production, efficient purification is required, which can be a major obstacle and a significant cost factor. The polyhistidine tag or His-tag is the most prominent tag for the purification of recombinant proteins [8]. Immobilized metal ion affinity chromatography (IMAC) is usually performed to separate His-tagged proteins from complex expression media on a suitable column [9]. IMAC is based on the interaction of the His-tag with metal ions that are bound by chelating ligands, mainly derived from NTA (nitrilotriacetic acid) or IDA (iminodiacetic acid) [10].

In contrast to other chromatographic separation methods, IMAC is a good compromise between capacity, effort, cost, and purity. Hence, it is evident that variants of IMAC will remain workhorses in the production of recombinant proteins for a long time to come. Finally, many vectors for the expression of recombinant proteins already contain His-tags, avoiding any changes in this respect.

In a recent paper, we presented protein-A-functionalized corundum particles for the purification of IgG from human plasma [11]. This protocol leads to products with high purity and offers a quick and easy protocol for the isolation of antibodies from complex samples. From these results, we concluded that functionalized corundum particles might also provide a suitable platform for His-tag-based purifications. The current work aimed to keep costs as low as possible and to facilitate the up-scaling of the protocol for any anticipated industrial application. These objectives suggest using low-cost silanes, such as APTES, since this substance is used in industry for all kinds of surface coatings [12,13]. EDTA dianhydride is applied as an economic intermediate for a new chelate ligand. Sánta-Bell et al. similarly used EDTA dianhydride, coupling it with aminoalkyl moieties for the immobilization of enzymes based on an IMAC interaction on porous polymeric beads [14]. Partchornik et al. successfully purified His-tagged proteins with BSA conjugates modified with EDTA dianhydride loaded with Cu^2+^ ions [15]. One of the active groups of EDTA dianhydride leads to acylation of the primary amine of the APTES, the other being hydrolyzed to carboxylic acids [16,17]. The base material, high-purity corundum, is at least 100 times cheaper (calculated per weight) than agarose beads.

In contrast to other reagents used in surface chemistry, EDTA dianhydride is quite an affordable chemical. The overall material cost of functionalized nickel–EDTA–corundum is less than 30 € cents per gram. Moreover, our approach only needs a standard centrifuge for separation instead of a more complex system for column-based chromatography [18]. In some cases, even simple sedimentation in a suitable flask might suffice.

Several standard protocols exist for purifications based on IMAC. This technique can also be performed in a “batch mode” [19], which is similar to the approach presented here. However, in this context, the designation “chromatography” might be misleading, and the more general term immobilized metal affinity separation (IMAS) may be preferable. Using a low concentration of imidazole (e.g., 20 mM) in the binding and washing buffer is usually recommended to suppress the unwanted binding of naturally occurring histidine-rich proteins of the hosts [20,21]. For elution, significantly higher imidazole concentrations (e.g., 250–500 mM) have to be applied [22]. Due to the use of an unusual chelate ligand in this work, the binding and elution conditions had to be adapted accordingly. In general, a compromise between capacity and product purity is recommended. In a direct comparison, the new corundum material was superior to the commercial Ni–NTA material tested in terms of purity and ease of application.

## 2. Materials and Methods

### 2.1. Materials

Corundum F1200 was obtained from Hasenfratz Sandstrahltechnik (Aßling, Germany). The vector pET-MBP-mSA2 was a gift from Sheldon Park (Addgene plasmid # 52319; http://n2t.net/addgene:52319; RRID:Addgene_52319) (accessed on 6 November 2022). pcDNA3-SARS-CoV-2-S-RBD-His8 was a gift from Erik Procko (Addgene plasmid # 145145; http://n2t.net/addgene:145145; RRID:Addgene_145145) (accessed on 2 May 2023). Other materials and reagents were obtained as follows: APTES 99% (CAS 919-30-2), dimethylformamide 99.9% (CAS 68-12-2), ethylenediaminetetraacetic dianhydride 98% (CAS 23911-25-3), imidazole 99% (CAS 288-32-4), LB medium (Miller) (8822-500G), lysozyme (62971-10G-F), nickel(II) sulfate hexahydrate 99% (CAS 10101-97-0), silver nitrate 99% (CAS 7761-88-8), tryptone, and yeast extract (9263-500G) were purchased from Th. Geyer GmbH & Co. KG (Renningen, Germany); bovine serum albumin (BSA) >98% (A7906), ethanol absolute, 99.5% (CAS 64-17-5), disodium hydrogen phosphate dihydrate (71643), dipotassium hydrogen phosphate ≥99.5% (60353), glutaraldehyde 50% in water (CAS 111-30-8), glycerol for molecular biology ≥99% (CAS 56-40-6), sodium dihydrogen phosphate dihydrate (71505), sodium chloride (71376), potassium dihydrogen phosphate anhydrous, ≥99.5% (60218), sodium dodecyl sulfate (CAS 151-21-3), NiCo21(DE3) *E. coli* cells (NEB, #C2529H), and BL21(DE3) pLysS *E. coli* cells (69451) were purchased from Sigma Aldrich (St. Louis, MO, USA); bromophenol blue (CAS 76-59-5), glucose monohydrate (1083422), ICP multi-element standard solution IV (1.11355), and Yttrium ICP standard (1.70368) were purchased from Merck KGaA (Darmstadt, Germany); glycine (CAS 56-40-6), PBS (1x Dulbecco’s)-Powder, isopropyl β-D-1-thiogalactopyranoside (IPTG) (APA1008.0025), and Tris base (CAS 77-86-1) were purchased from AppliChem GmbH (Darmstadt, Germany); protein-A/G His-tagged (PAG-His6) and recombinant >96% PRO-1927 were purchased from Prospec-Tany Technogene Ltd. (Ness-Ziona, Israel); kanamycin (26899.03), Serva Dual Color protein standard III, and Quick Coomassie Stain (35081.01) were purchased from Serva Electrophoresis GmbH (Heidelberg, Baden-Württemberg Land, Germany); Pierce BCA Protein Assay Kit (23225), sodium cyanoborohydride 95% (CAS 25895-60-7), and trifluoroacetic acid 99.5% (TFA, 85183) were obtained from Thermo Fisher (Waltham, MA, USA); protease inhibitor cOmplete EDTA-free (4693159001) was purchased from Roche (Mannheim, Germany); Ni–NTA agarose beads (AC-501-10) were purchased from JenaBioScience (Jena, Germany); RO (reversed osmosis) water was used from a Milli-Q water purification system (Millipore, Bedford, MA, USA) with a resistivity of >18.2 Ω and TOC value of <5ppm.

### 2.2. Functionalization of Corundum

Before functionalization, the raw corundum powder was purified with a protocol recently described in [11]. In our recent work, we observed needle-shaped impurities on the corundum surface, containing sodium and chlorine. We could show that the following treatment eliminates these completely. In brief, 1 g of corundum was washed with 5 mL of 10% KOH solution for 10 min at RT before 5 mL of 37% hydrochloric acid was added and transferred to hydrolysis glass tubes. Next, the sample was heated to 100 °C for 20 min. After dilution in 1:2 lab water, the dispersions were washed thoroughly with 5 mL lab water three times before drying under a vacuum overnight. During the washing steps, the discarding of fine particles might be considered.

An amount of 1 g of corundum was incubated with 5 mL of 10% APTES solution in 99:1 ethanol:lab water at room temperature (RT) for 16 h in an overhead rotator at 20 rpm. After washing three times with ethanol, 1% EDTAD in DMF (50 mg in 5 mL DMF) was incubated for 30 min at RT. After centrifugation (6000 rpm, 5 min), the supernatant was discarded, and a second incubation step with 1% EDTAD occurred for 30 min. Three washing steps with DMF followed before incubation with 2.5 mL of nickel(II) sulfate hexahydrate (160 mM) was performed for 30 min at RT.

### 2.3. Kaiser Test

Three solutions were used to perform the Kaiser test [23] for the determination of free primary amine groups: solution A containing 250 mg of ninhydrin in 5 mL of ethanol and solution B containing 4 g of phenol in 5 mL of ethanol. An amount of 6.5 mg of KCN (potassium cyanide) was dissolved in 10 mL of lab water. In order to obtain solution C, 200 µL from that solution was further diluted in 10 mL of conc. pyridine. A spatula tip from each particle sample was transferred into a tube and incubated with a mixture of the solutions A, B, and C, an equal volume of each (here, 20 µL). The tubes were heated to 90 °C for 5 min.

### 2.4. ICP-MS Determination

For the determination of the nickel content, 1 g of Ni-loaded corundum and a blank sample that had not been loaded with Ni was dispersed in 2 mL of 65% HNO_3_ and then heated to 100 °C for 1 h. These harsh conditions released all the nickel ions into the solution. After cooling and sedimentation of the corundum particles, the sample was diluted 1:33 with lab water and centrifuged for 5 min at 6000 rpm. Nickel concentrations were measured on a Thermo iCAP Q using the multi-element standard IV from Merck for external calibration and applying yttrium (Y) as the internal standard.

### 2.5. Optimization of Imidazole Content in Binding Buffer

An amount of 0.5 g functionalized corundum was incubated with 2.5 mL of protein solution containing 1 mg of PAG and 5 mg of BSA. For each sample, the imidazole content varied from 5 to 40 mM imidazole in the binding buffer (TBS) (2.5 mL, pH 7.2, containing: 0.136 M NaCl, 0.0027 M KCl, 0.099 M Tris base). After the addition of imidazole, the pH was adjusted with 37% HCl. The imidazole concentration in the Tris buffer is given as follows: TBS40 for 40 mM imidazole, TBS20 for 20 mM imidazole, and so on. After incubation in an overhead rotator at 20 rpm for 1 h at RT, the particles were washed three times with TBS. The elution of bound protein was performed with 2.5 mL TBS elution buffer containing 500 mM imidazole for 30 min at RT in an overhead rotator. All eluates were collected, and from each eluate, 18 µL was added to 6 µL of SDS loading buffer (see Section 2.6).

### 2.6. SDS-PAGE

As previously described [11], SDS-PAGE was performed with the electrophoresis system XCell SureLock from Invitrogen AG (Waltham, MA, USA) with a Novex Tris-Glycine Mini Gel (8 to 16%, XP08160BOX). SDS loading buffer contained (4× concentrated: Tris base (252 mM, pH 6.8), glycerol (40%), SDS (8%), and bromophenol blue (0.02%) in lab water). In total, 18 µL of the sample was added to 6 µL of 4× SDS loading buffer. The samples were heated to 90 °C for 5 min and cooled down to RT before being loaded onto the gel. Electrophoresis was performed at 70 V for 10 min and 180 V for another 60 min. For staining, the Quick Coomassie Stain (35081.01 Serva) was used according to the manufacturer’s description before the gel was stained with silver. The aqueous silver staining solutions (150 mL each) were prepared as follows: (1) 0.02% sodium thiosulfate (water-free, 98%), (2) 0.1% silver nitrate, (3) 2.5% sodium carbonate, (4) 2.5% sodium carbonate and 0.02% formaldehyde, and (5) 50 mM EDTA disodium salt. The Coomassie-stained gel was incubated with solution (1) for 1 min and washed twice with lab water. The incubation with solution (2) took place for 25 min before washing once with lab water. Then, solution (3) was incubated for 10 s before the solution was discarded and solution (4) was added without any washing in between. The gel was stained for 10 min before another washing step, and the incubation with solution (5) took place for 5 min. The gel was then finally washed with lab water.

### 2.7. BCA Assay for the Determination of Protein Binding Capacity

In order to determine the protein binding capacity on nickel-loaded corundum, 100 mg corundum per sample was incubated with different amounts of His6-PAG ranging from 0 to 600 µg in 100 µL TBS containing 40 mM imidazole (TBS40). After the incubation for 1 h at RT, supernatants were collected after centrifugation for 5 min at 13,000 rpm. The supernatants were then analyzed with a BCA assay according to the protocol from Pierce BCA Protein Assay Kit (MAN0011430). An amount of 25 µL per supernatant and PAG-His6 standard solution were pipetted into a microplate well before 200 µL of the working reagent containing BCA reagents A and B (50:1) was added. The microplate was then incubated for 30 min at 37 °C. The absorbance was measured at 562 nm on the microplate UV-Vis reader BioTek Epoch 2 with the software Gen 5 (Version 2.07, BioTek Instruments, Inc., Winooski, VT, USA).

### 2.8. Production of His6-MBP-mSA2 and Cytoplasm Blanks in Different E. coli Strains

As a real-life sample, His6-MBP-mSA2 (fusion protein consisting of monomeric streptavidin and maltose-binding protein) was expressed to be later purified by functionalized corundum. A detailed description of the expression can be found in the Supplementary Material, Section S6. Two different *E. coli* strains were used, BL21(DE3) pLysS and NiCo21(DE3). 

### 2.9. Production of SARS-CoV-2-S-RBD-His8 Domain

In order to investigate another recombinant protein in a complex matrix, the plasmid for the expression of the His8-SARS-CoV-2-S-RBD-His8 domain was transfected into Expi293F-cells. A detailed description of the expression can be found in the Supplementary Material, Section S7.

## 3. Results and Discussion

### 3.1. Functionalization of Purified Corundum

**Figure 1:**
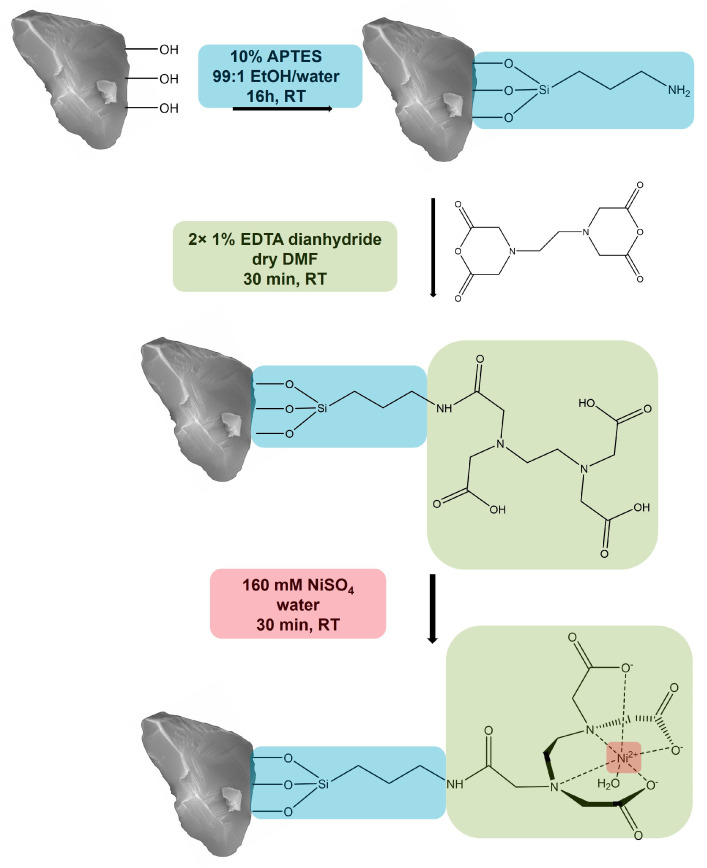
Functionalization of corundum with APTES, EDTA dianhydride, and Ni^2+^ to purify His-tagged proteins (schematic representation).

We created a new corundum-based platform acting similarly to an ion metal affinity chromatography (IMAC) support. The first functionalization step was the covalent attachment of the common amino silane APTES to obtain an amine function on the corundum surface. Previous experiments with this silane indicated that 10% of APTES at room temperature and a reaction time of 16 h led to higher binding yields compared to 1%. After silanization, a solution of 1% EDTA dianhydride in dry DMF was added. This fast reaction will lead to an amide bond between the amine group of the APTES and a ring opening of the anhydride, resulting in the covalent attachment of EDTA to the corundum surface. When incubated with a solution of nickel ions, this ligand will form a complex with Ni^2+^, which will later interact with the Histidine residues of the His-tagged protein that is desired to be purified. The described functionalization scheme is shown in Figure 1.

**Figure 2:**
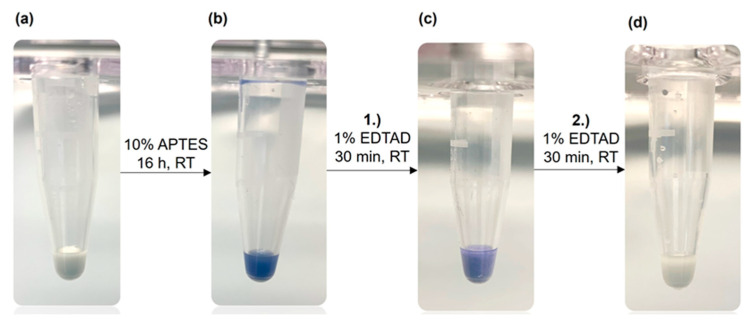
Kaiser test (ninhydrin-based) for the detection of primary amine groups on the corundum surface. (**a**) Unmodified corundum showing no blue color, (**b**) strong blue color after functionalization with APTES, (**c**) a weaker blue color after the first incubation with EDTA dianhydride (EDTAD), and (**d**) no blue color after the second incubation with EDTA dianhydride, indicating that all primary amines have been derivatized.

To verify the surface modification described above, we applied the Kaiser test. This is a very sensitive assay for the qualitative determination of primary amine groups [24,25] frequently applied in peptide synthesis. First, the Kaiser test can confirm that the silanization with APTES was successful. In the next step, the reaction with EDTA dianhydride, the faint blue color indicates that the derivatization is still incomplete. Figure 2 illustrates the Kaiser test with unfunctionalized corundum, which does not show any blue color (a). After a successful reaction of corundum with APTES, a strong blue color was observed with the Kaiser test (b), confirming the amino silanization of the surface. After incubation with 1% of EDTA dianhydride in dry DMF for 30 min (c), the blue color was weaker, indicating that some non-reacted primary amine groups were still present. After a second incubation with 1% EDTA dianhydride solution (d), no blue color could be detected with the subsequent Kaiser test, meaning that the derivatization is completed.

### 3.2. Determination of Nickel Content via ICP-MS

**Figure 3:**
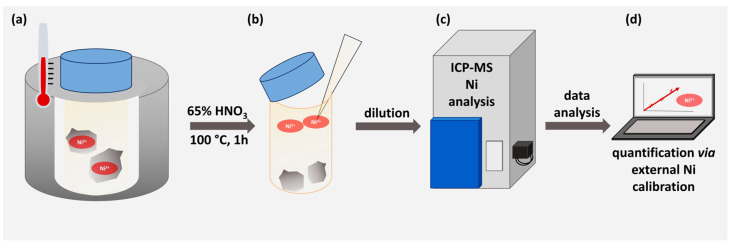
Workflow for the nickel determination via ICP-MS. (**a**) Unmodified and modified corundum particles were treated with 65% HNO_3_ for 1 h at 100 °C. (**b**) The strong acid was diluted 1:33 with pure lab water. (**c**) ICP-MS determination was performed. (**d**) An external calibration was used to quantify the nickel concentration.

In order to quantify the nickel capacity of the modified material, 1 g of functionalized and nickel-loaded corundum as well as 1 g of Ni-unmodified corundum as a negative control were treated with concentrated HNO_3_ to release the metal ions into the supernatant. This direct approach can be considered very reliable and highly quantitative [26]. Figure 3 illustrates the working scheme.

The ICP-MS results are shown in Table 1. The nickel content of fully functionalized corundum particles was more than 3500 times higher than in the blank samples. This underlines the successful functionalization and proves the high nickel capacity of the surface.

If a 2:1 molar binding ratio of Ni^2+^ with a His-tagged protein of ca. 66 kDa (such as BSA) is assumed [27], the nickel value would theoretically allow a bound protein mass of about 170 mg protein/g corundum. This means that the amount of immobilized nickel is in vast excess relative to the protein and does not limit the capacity. This result also suggests that polyhistidines may bind to two or more nickel ions [28], without the need for multivalent chelators. It is impossible to exploit the full capacity of the nickel ions with much bigger molecules, such as a protein, due to steric reasons. This corundum material has a surface area of around 4.5 m^2^/g [11]. According to [29], a side-on interaction of BSA on 1 cm^2^ corresponds to 223 ng BSA. Therefore, for a complete protein monolayer on 1 g corundum, about 10 mg of BSA would be needed. Therefore, this would be the maximum theoretical capacity for BSA and other proteins of similar size.

### 3.3. Optimization of Imidazole Concentration in Binding Buffer

**Figure 4:**
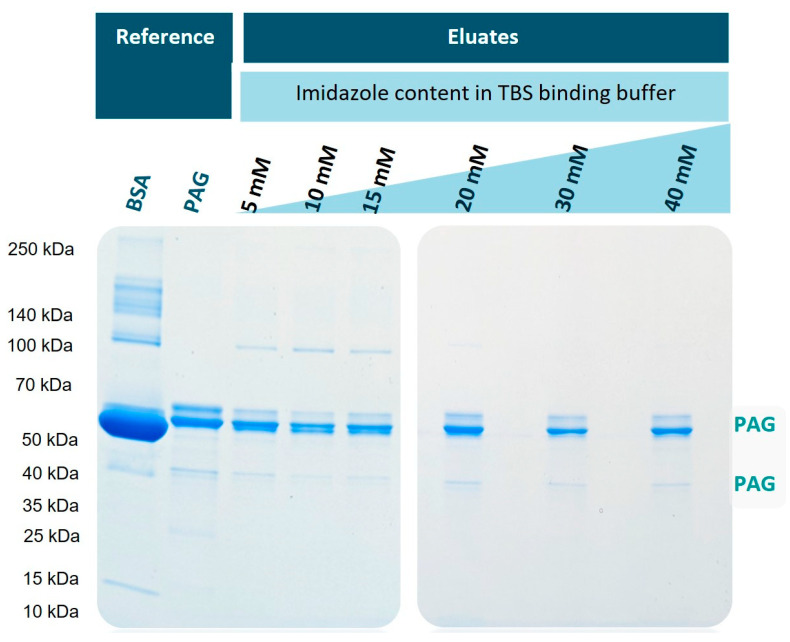
Variation in the imidazole concentration in the binding and washing buffers (TBS); SDS-PAGE electrophoresis of separated His6-PAG from a BSA-containing solution. An imidazole concentration of 20 mM and higher led to the purest preparations, with slightly decreasing yields. The bands caused by the PAG preparation are marked on the right side of the figure.

In order to examine the potential of the new corundum platform for the purification of proteins, His-tagged protein A/G (His6-PAG) was separated from a mixture with bovine serum albumin (BSA). Having 17 histidines in its structure [30], BSA can be a potential candidate to interact with the Ni^2+^ on the corundum surface to a certain extent. In general, it can be assumed that the higher the imidazole content during the binding step, the higher the selectivity will be, with the potential drawback that the capacity may decrease and some nickel leaching might occur. BSA and His6-PAG were mixed in a mass ratio of 5:1 to explore the selectivity of this method. Figure 4 shows the SDS-PAGE separation of the eluates obtained after the elution with 500 mM imidazole buffer. The used BSA was of technical quality; hence, it was no surprise that several protein bands were visible in the reference lane (lane 1). The recombinant His6-PAG also shows some impurities and heterogeneities, most prominent in the double bands with the highest intensities (lane 2). When imidazole was used in the binding buffer, the potential interaction of BSA and its impurities should be inhibited in a concentration-dependent fashion. This becomes evident with increasing imidazole content in the binding (and washing) buffers. When 5–15 mM imidazole was used (lanes 3–5), some protein bands from BSA were still visible. This effect is reduced when 20 mM or higher concentrations of imidazole are used (lanes 6–8).

For further experiments, the imidazole content of 20 mM was chosen as a binding buffer since the eluate showed few impurities with high protein capacity. Therefore, when having real-life samples, a binding buffer with 40 mM imidazole was prepared and then diluted with the sample 1:2 to have a final imidazole concentration of 20 mM. This TBS40 was also used as a washing buffer. When the highest protein binding capacities are desired, a lower imidazole content could be beneficial. For these applications, a 10 mM imidazole binding buffer (TBS10) was also tested in further experiments.

### 3.4. BCA Assay for the Determination of Protein Binding Capacity in TBS20 and TBS40

**Figure 5:**
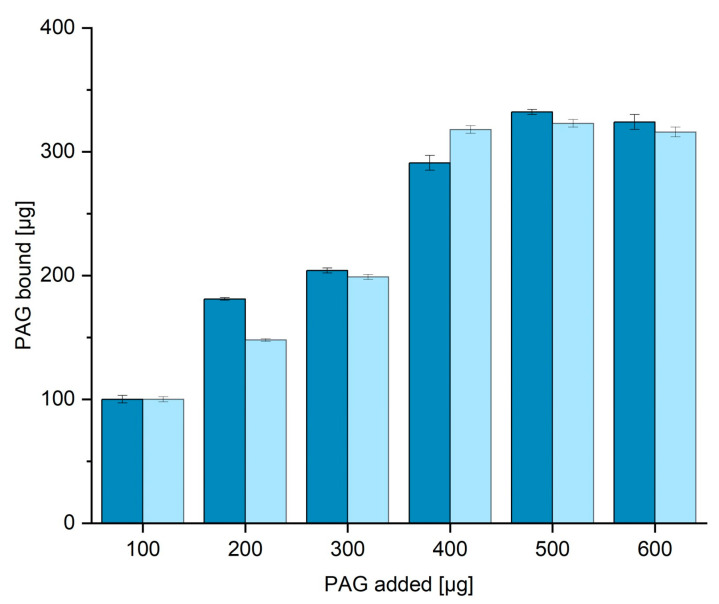
Determination of the protein binding capacity (binding buffer TBS20 dark blue, TBS10 light blue). The amount of bound His6-PAG is calculated by subtraction of the residual amount from the amount added. A His6-PAG calibration curve (BCA) is shown in the Supplementary Material (Figure S2). In addition, it could be shown that the use of 40 mM imidazole instead of the concentration of 20 mM does not significantly reduce the protein binding capacity of the material.

The binding capacity of nickel-loaded corundum for His6-PAG was determined with 100 mg aliquots. First, 100 mg of functionalized corundum was incubated with different amounts of His6-PAG ranging from 0 to 600 µg. The incubation took place for one hour at RT in an overhead rotator before the supernatants were collected and measured with the BCA assay [31]. Figure 5 shows the amount of PAG bound to the surface when the amount in the supernatant is subtracted from the initial PAG content. When incubated with more than 400 µg PAG, around 300 µg can be bound to the surface. This means 1 g of functionalized corundum can bind up to 3 mg of His6-PAG (52 nmol), or 2.4 mg per mL of corundum suspension. This value is in the same range as the protein binding capacities of corundum determined in our previous work, where the bound protein was quantified directly on the corundum surface by amino acid analysis followed by HPLC quantification [11]. No significant difference in capacity was obtained when different binding buffers with even higher amounts of imidazole up to 40 mM were used.

### 3.5. Purification from E. coli Cell Lysates Spiked with His6-PAG

**Figure 6:**
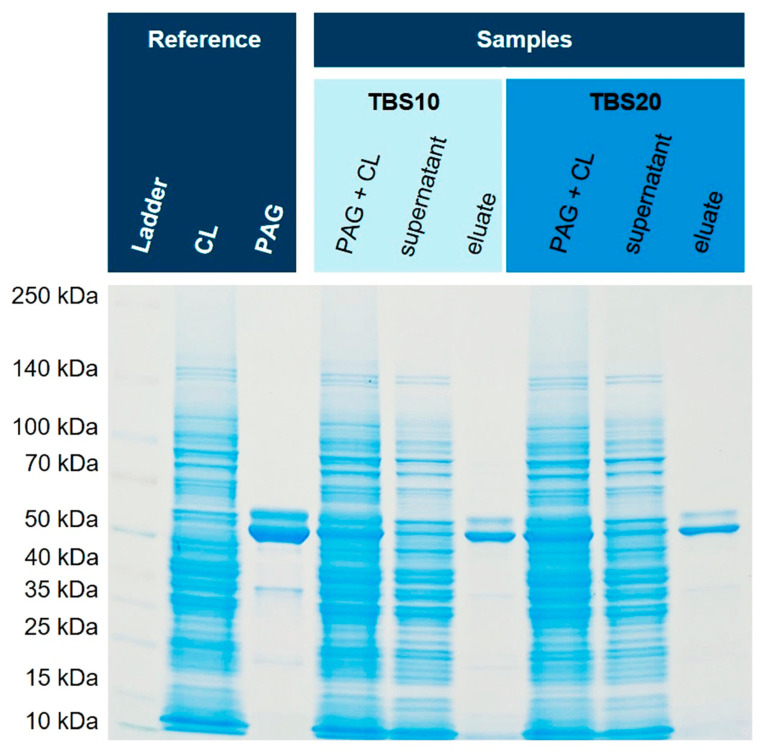
Isolation of His6-PAG from spiked *E. coli* NiCo21(DE) cell lysate (CL). For both binding buffers (TBS10 and TBS20), the isolation of His-tagged PAG was successful with high purity. A silver-stained gel is shown in the Supplementary Material (Figure S3).

In order to test matrices of high complexity, the soluble fraction of *E. coli* cell lysates of two different strains, NiCo21(DE) and BL21(DE) pLysS, containing endogenous histidine-rich proteins, were spiked with His6-PAG. *E. coli* cytoplasm can contain up to 320 mg/mL of total protein [32] and is a demanding matrix for the selective purification of a single protein. First, the spiked NiCo21(DE) sample was diluted 1:2 in TBS20 or TBS40, leading to a final imidazole concentration of 10 mM or 20 mM in the extraction solution, respectively. After incubation, three washing steps with a buffer containing either 20 mM (for the 10 mM sample) or 40 mM (for the 20 mM sample) imidazole were performed. Finally, the bound His6-PAG was dissociated from the corundum with 500 mM imidazole for 30 min for both samples. Figure 6 shows the obtained SDS-PAGE gels with reference and sample lanes.

**Figure 7:**
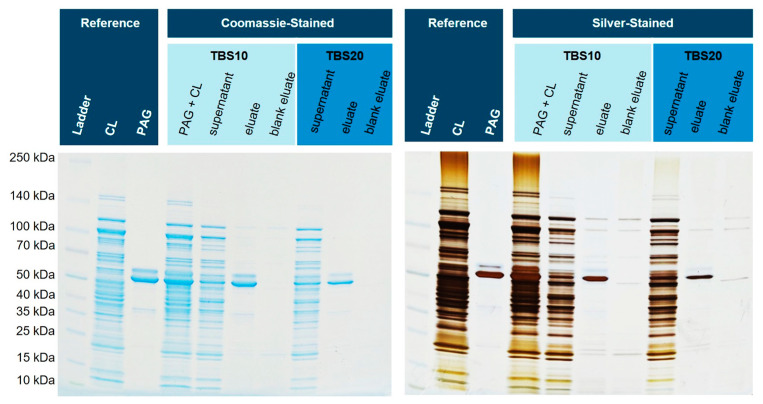
Isolation of His6-PAG spiked into the cell lysate (CL) of *E. coli* BL21(DE3)pLysS. The silver-stained gel reveals that the matrix eluate shows fewer impurities when using TBS20 as a binding buffer.

Both binding and washing buffers show very similar isolation and purification characteristics of His6-PAG from the cell lysate of NiCo21(DE), an *E. coli* strain with reduced endogenous histidine-rich proteins. In this specific case, GlmS (Glutamine-fructose-6-phosphate aminotransferase) is mutated to reduce co-binding [33]. The purity of both eluates is excellent, and the intensity of the bands is similar. Therefore, we concluded that an imidazole concentration of 10 mM in the binding and 20 mM in the washing buffer seems suitable to avoid non-specific binding and does not compromise capacity. Subsequently, we tested this method with an *E. coli* strain with a normal content of endogenous histidine-rich proteins that could interfere with affinity purification, and hence, His6-PAG was spiked into the cytoplasm originating from *E. coli* BL21(DE3)pLysS. Figure 7 shows the corresponding SDS-PAGE gel. Next to the spiked eluates, a matrix eluate was examined, where no His6-PAG was added. These lanes might show any non-specific interactions of endogenous proteins in more detail.

The eluate with a binding buffer containing 10 mM imidazole shows a slightly higher number of blank proteins than the buffer with 20 mM. Furthermore, it can be shown that most of the His6-PAG is bound and eluted with TBS10. With higher concentrations of imidazole in the binding and washing buffer, the amount of bound His6-PAG decreased, but the purity was improved. This effect can also be seen in the blank eluates (cytoplasm without His6-PAG).

### 3.6. Enrichment of His6-PAG from Higher Volumes

**Figure 8:**
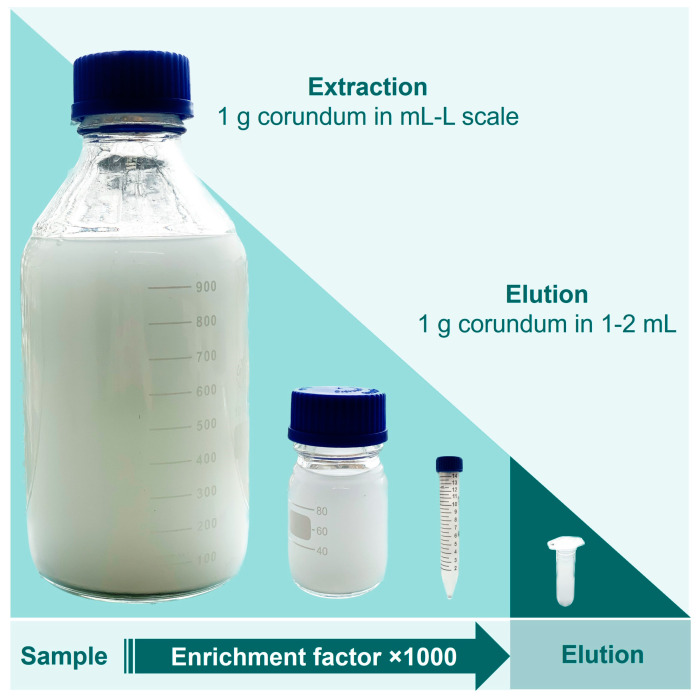
Reduction of a sample volume between 100 and 1000 mL resulting in a final elution volume down to 1 mL (schematic representation).

**Figure 9:**
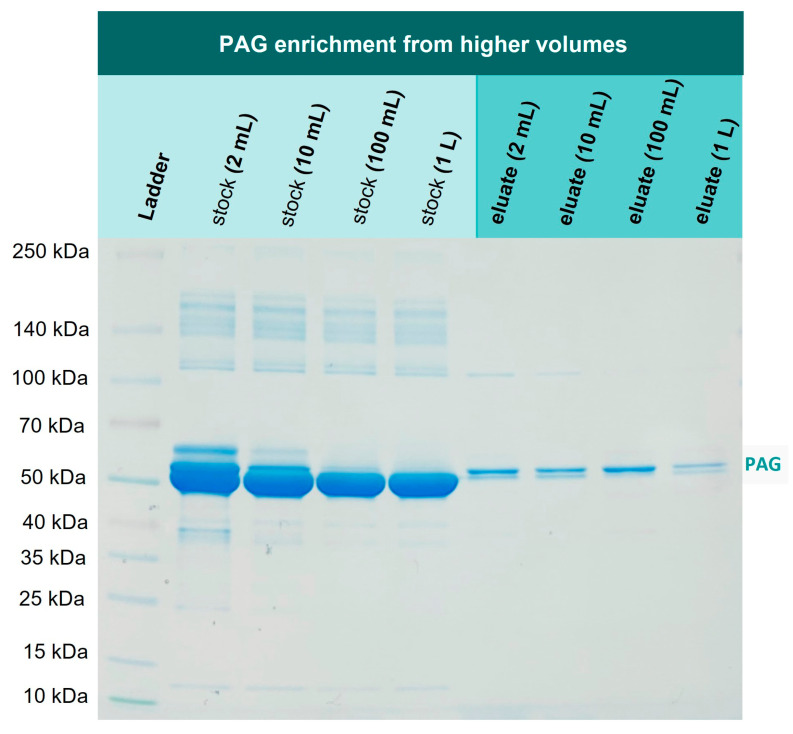
Enrichment experiment: 1 mg of His6-PAG was spiked into different volumes (2–1000 mL) of 0.1% BSA in TBS10. His-tagged protein could be enriched in one step even from highly diluted solutions. The double band marked at the right side of the figure is caused by the PAG preparation.

In the next experiment, we investigated if 1 mg PAG can be re-isolated from higher volumes as well, addressing the issue of a one-step recombinant protein purification that is performed on larger scales and is still considered challenging [34]. For this purpose, 1 mg PAG was spiked into 2 mL, 10 mL, 100 mL, and 1000 mL of TBS10 and incubated with 1 g Ni-loaded corundum, each. The incubation took place overnight in an overhead rotator. All samples were washed three times with TBS10 and then eluted with 500 mM imidazole. Figure 8 illustrates the mentioned volumes to visualize the reduction of the sample volume from extraction to elution, resulting in a significant protein enrichment. Figure 9 shows the correspondent SDS-PAGE of the extraction and elution solutions.

Lanes 2–5 in Figure 9 show the corresponding stock solutions of 1 mg His6-PAG in different volumes of a 0.1% BSA solution as a matrix model. The His6-PAG concentration in the stock solutions decreases with higher volumes to a point where the His6-PAG band in 1000 mL is not even visible, whereas the matrix concentration was kept constant. In contrast, in the eluate of the 1000 mL sample, the His6-PAG band is clearly visible, indicating successful enrichment from a higher volume. The amount of His6-PAG in that sample was 1000 times lower than BSA, which is also corresponding to a selectivity factor of 1000. This is very promising in terms of up-scaling the purification process, which due to the low costs for the corundum material, is considered as realistic. When an enrichment from even higher volumes is desirable, it must be taken into consideration that the affinity of Ni chelates to bind His-tags might not be sufficient. Choosing tags with higher affinities could be a way to counteract this issue.

### 3.7. Comparison of Corundum Platform with Commercial Ni–NTA Agarose Beads

**Figure 10:**
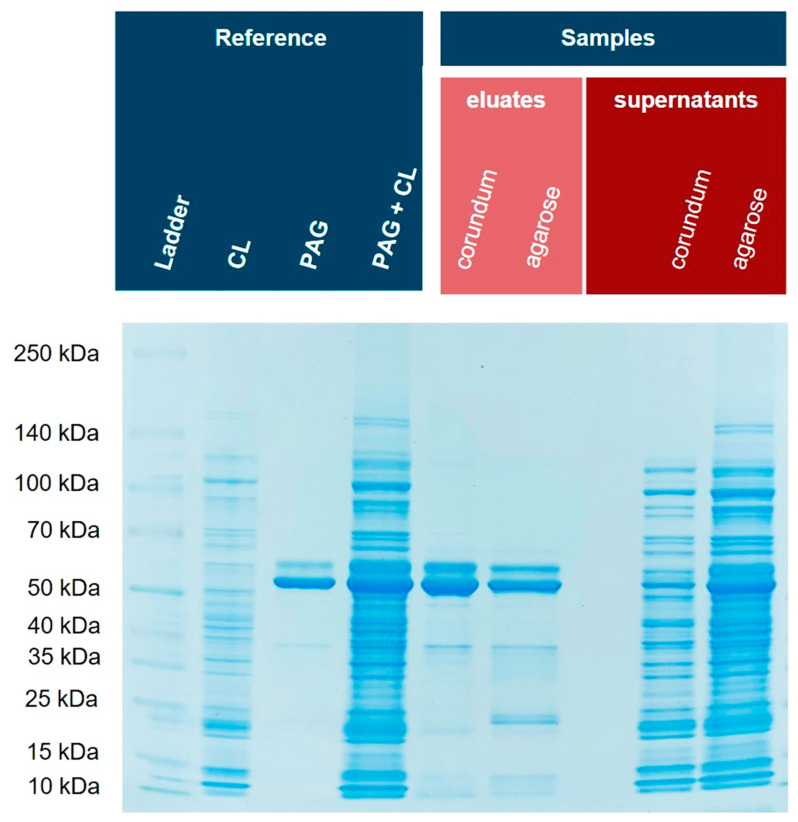
Isolation of His6-PAG from spiked *E. coli* cell lysate by nickel-loaded corundum particles compared with a commercial Ni–NTA agarose material. Similar intensities of the respective bands in the PAG + CL and corundum eluate lanes show that PAG could be nearly quantitatively bound from the sample. This notion is also supported by the supernatants. CL: cell lysate, PAG: His6-tagged protein A/G.

Next, we investigated how the corundum particles perform compared to well-established and commercial Ni–NTA agarose beads. Again, His6-PAG was spiked into *E. coli* BL21(DE3)pLysS cell lysates and the samples were purified with the established corundum method as well as Ni–NTA agarose beads according to the manufacturer’s protocol (Supplementary Material). Different buffers and volumes were used for both protocols to compare both methods under their optimized conditions. For corundum, a TBS20 for binding and a TBS40 for washing were used, and finally, an elution buffer with 500 mM imidazole. The standard agarose protocol was performed with 10 mM imidazole in the binding buffer, 20 mM imidazole in the washing buffer, and elution was performed with 250 mM imidazole. Figure 10 shows the respective SDS-PAGE gel with a Coomassie stain. The eluate of the corundum particles contains fewer impurities than the nickel–NTA agarose beads (particularly in the lower molecular weight range).

### 3.8. Purification of Recombinant His6-MBP-mSA2 Fusion Protein from E. coli Cytoplasm

**Figure 11:**
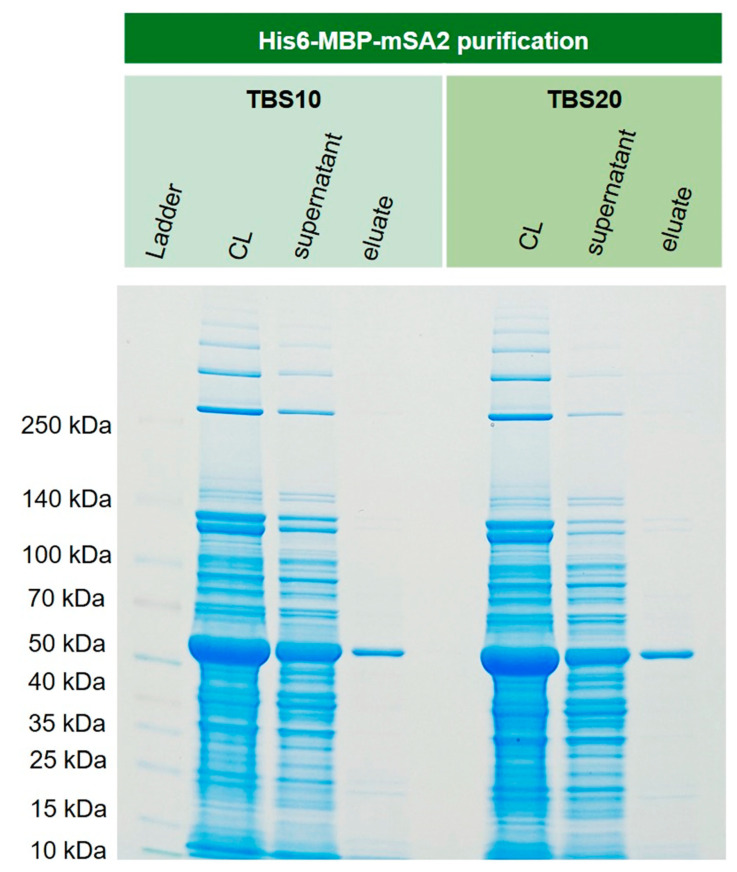
Extraction of cytoplasmically expressed His6-MBP-mSA2 (Maltose binding protein and monovalent streptavidin tagged with N-terminal His6) from *E. coli* BL21(DE3)plysS. The extraction and purification of the fusion protein were successful for TBS 10 and TBS20. No significant difference could be observed for these binding buffers.

In order to show that this new corundum material also performs well with real-life samples, recombinantly expressed His6-MBP-mSA2 was extracted from *E. coli* cell lysate. This protein, with a mass of around 60 kDa, could be isolated in one step with excellent purity. The respective SDS-PAGE gel is shown in Figure 11. The lane of the supernatant revealed an overload in the capacity of the material. This is a general issue in affinity methods if the approximate concentration of the target is unknown in advance.

### 3.9. Purification of Recombinant SARS-CoV-2-S-RBD-His8 from Expi293F Cells

**Figure 12:**
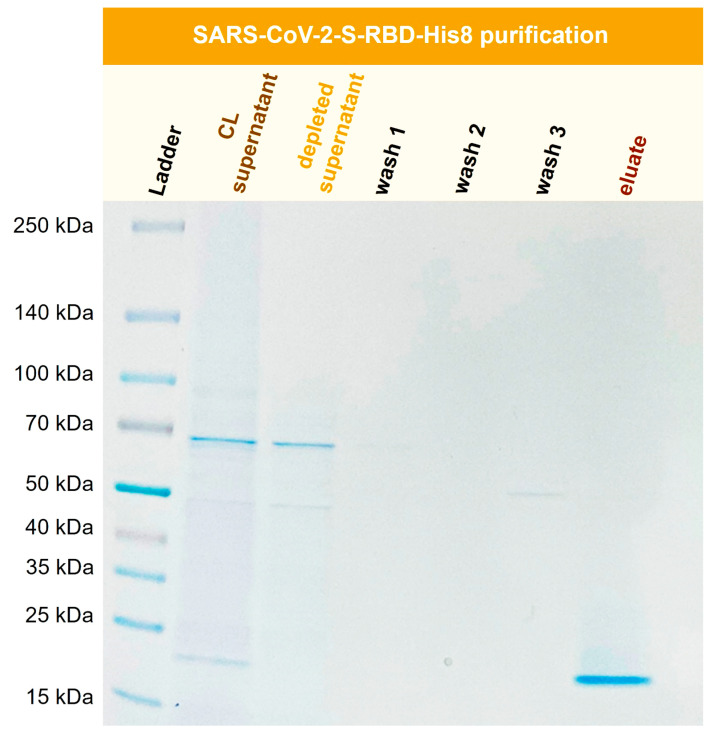
Purification of recombinant SARS-CoV-2-S-RBD-His8 (23.4 kDa) from Expi293F cell supernatant (CL). TBS20 was used as the binding buffer and TBS500 for elution.

The SARS-CoV-2 pandemic greatly increased interest in virus-related proteins and their recombinant expression. The SARS-CoV-2 spike protein (S) interacts with a human cell surface receptor and is crucial for infection [35]. It can be expressed in cell cultures as a valuable tool for ELISA and LFA assay development or vaccine development [36]. Here, a recombinant SARS-CoV-2 S-RBD (receptor-binding domain) was purified with Ni-functionalized corundum from a human Expi293F cell culture supernatant. Figure 12 shows the SDS-PAGE gel for the purification process of SARS-CoV-2-S-RBD-His8 from Expi293F cell culture supernatant with a molecular mass of 23.4 kDa.

Figure 12 shows the successful isolation and enrichment of a recombinant receptor-binding domain (RBD). No product is visible in the depleted supernatant, indicating that the protein was extracted quantitatively from the cell culture (CL) supernatant. The wash solutions show little to no protein. The eluate consists of the His-tagged RBD domain in high purity.

## 4. Conclusions

High-purity corundum powder was used in this work to develop a new affinity purification platform based on the well-known interaction between nickel ions and polyhistidine tags. ICP-MS confirmed successful surface modification and nickel-loading. Even protein purification from the cell lysates of non-optimized *E. coli* strains delivered the expected yields and excellent purities of the recombinant target proteins. In direct comparison, the eluates of the corundum protocol showed fewer impurities than a standard protocol based on nickel–NTA agarose beads by a commercial vendor. As real-life examples, the recombinantly expressed fusion protein His6-MBP-mSA2 and SARS-CoV-2-S-RBD-His8 were successfully purified from *E. coli* and human cell culture medium, respectively. Due to the column-free approach, up-scaling seems straightforward, supported by the very low cost of the corundum material. This could overcome the limited application of immobilized metal affinity separation in industry. Other advantages of corundum, some of which have been discussed before [11], are the availability of many different particle sizes, extreme physical and chemical stability, very fast binding and elution kinetics due to a lack of pores, the flexibility of the surface chemistry, and the option for enrichment, which makes additional concentration steps often superfluous. A disadvantage of corundum is its capacity, calculated by volume or weight, due to the absence of internal pores. However, this can be easily compensated by a higher amount of this cheap material. In addition, the processing of larger quantities of low-concentration supernatants is possible. Furthermore, suspended matter cannot lead to any pressure issues, such as those frequently encountered in column-based affinity methods. Finally, because of its extremely low cost, corundum can be considered disposable, bypassing the tedious regeneration and decontamination of the material. Hence, capacity losses during regeneration, carryover, and disinfection are not an issue. Due to the superior chelate structure in comparison to the standard NTA-based materials, the leaching of nickel ions should be minimized.

## Figures and Tables

**Table 1:** Nickel determination via ICP-MS of loaded corundum and EDTA-functionalized corundum as a negative control. The nickel calibration curve is shown in the Supplementary Material (Figure S1).

Sample	Ni µmol/g Corundum	Ni^2+^ µg/g Corundum
Nickel-loaded corundum	5.2 ± 0.1	304 ± 6
Blank material	0.0015 ± 0.0001	0.09 ± 0.01

## Data Availability

Not applicable.

## Supplementary Materials

The following supporting information can be downloaded at: https://www.mdpi.com/article/10.3390/biotech12020031/s1; Figure S1: External Ni calibration for the Ni determination of functionalized corundum via ICP-MS using a Thermo iCAP Q and the multi-element standard for external calibration and yttrium as internal standard. Figure S2: His6-PAG calibration for BCA-Assay. His6-PAG was diluted in different concentrations for external calibration. The absorbance was measured at 562 nm. Figure S3: Silver-stained gel for the isolation of His6-PAG-spiked *E. coli* NiCo21(DE3) cell lysate. For both binding buffers containing 20 mM and 40 mM imidazole, no difference in purity is presently confirming that with a cytoplasm optimized for IMAC, the use of 20 mM imidazole as a dilution buffer is not compromising purity. Figure S4: Determination of the protein binding capacity (binding buffer TBS40 dark blue, TBS20 light blue). A maximum of around 300 mg His6-PAG in the supernatant was determined when incubating 100 mg functionalized corundum with 600 µg PAG.
