# Supplementary material for: Efficient Purification of Polyhistidine-Tagged Recombinant Proteins Using Functionalized Corundum Particles
Source: BioTech (Basel). 2023 May 3;12(2):31. doi: 10.3390/biotech12020031 (PMC10204482; doi:10.3390/biotech12020031)
Supplement: Supplementary file 1 [file biotech-12-00031-s001.zip › biotech-2357737-supplementary.pdf]

BioTech

Supplementary Material

Efficient Purification of Polyhistidine-Tagged Recombinant Proteins Using Functionalized Corundum Particles

Jule L. Völzke, Sarah Smatty, Sarah Döring, Shireen Ewald, Marcus Oelze, Franziska Fratzke, Sabine Flemig, Zoltán Konthur, and Michael G. Weller*

Federal Institute for Materials Research and Testing (BAM), Richard-Willstätter-Strasse 11, 12489 Berlin, Germany
*Correspondence: michael.weller@bam.de;
ORCID: 0000-0003-2767-2029, Tel.: +49-30-8104-1150

Citation: Völzke, J.L.; Smatty, S.; Döring, S.; Ewald, S.; Oelze, M.; Fratzke, F.; Flemig, S.; Konthur, Z.; Weller, M.G. Efficient Purification of Polyhistidine-Tagged Recombinant Proteins Using Functionalized Corundum Particles. *BioTech* **2023**, *12*, 31. https://doi.org/10.3390/biotech12020031

Received: 5 April 2023

Revised: 26 April 2023

Accepted: 28 April 2023

Published: 3 May 2023

Publisher's Note: MDPI stays neutral with regard to jurisdictional claims in published maps and institutional affiliations.

Copyright: © 2023 by the authors. Submitted for possible open-access publication under the terms and conditions of the Creative Commons Attribution (CC BY) license (https://creativecommons.org/licenses/by/4.0/).

1. ICP-MS – Nickel calibration

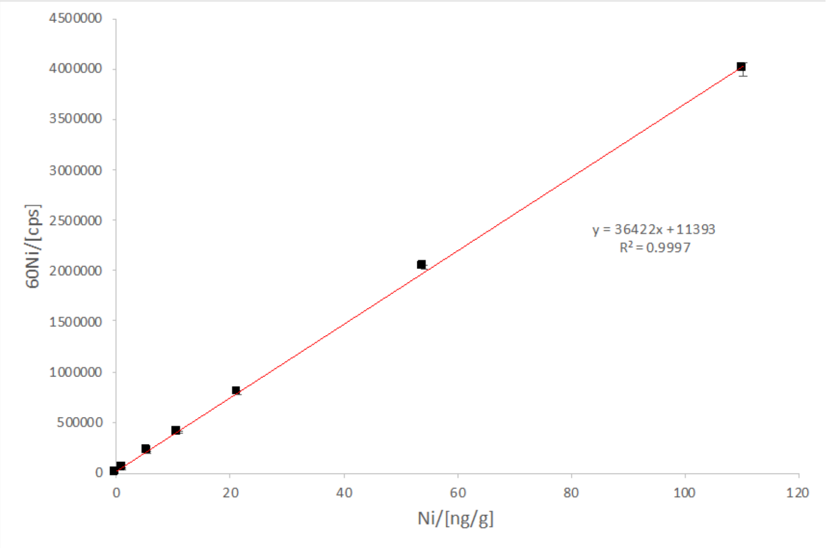

Figure S1. External Ni-calibration for the Ni-determination on functionalized corundum via ICP-MS using a Thermo iCAP Q and the multi-element standard for external calibration, and yttrium as internal standard.

2. His6-PAG calibration by BCA protein assay

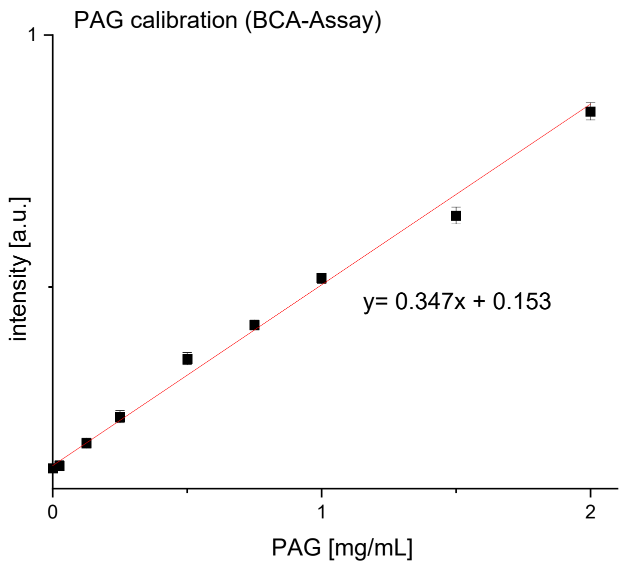

Figure S2. His6-PAG calibration by BCA protein assay. His6-PAG was diluted in different concentrations for external calibration. The absorbance was measured at 562 nm.

3. Isolation of His6-PAG spiked into *E. coli* NiCo21(DE3) cell lysate – Silver-stained gel

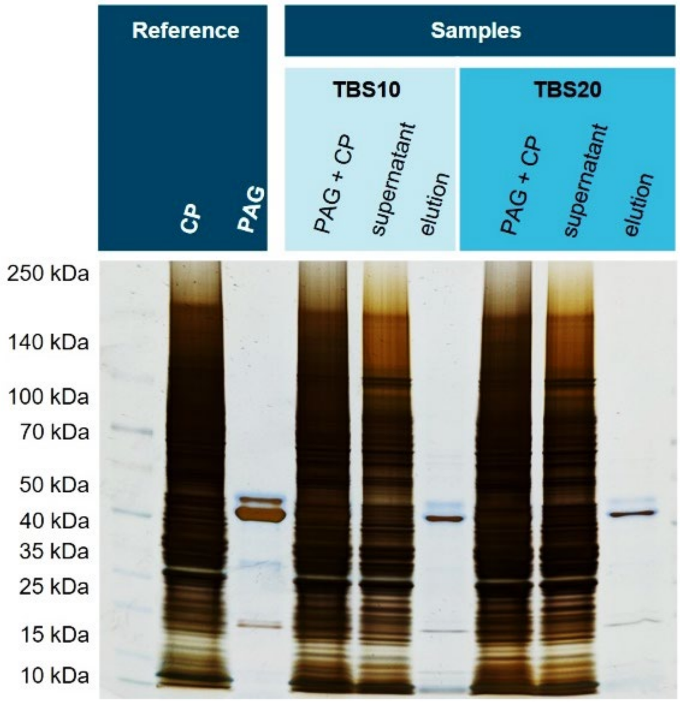

Figure S3. Silver-stained gel showing the isolation of His6-PAG spiked into *E. coli* NiCo21(DE3) cell lysate**.** For both binding buffers TBS 10 and TBS20, there seems to be no difference in purity. This confirms that for a cytoplasm optimized for IMAC, the use of 20 mM imidazole as a dilution buffer does not compromise selectivity.

4. BCA assay for the determination of protein binding capacity in TBS20 and TBS40 in the supernatant

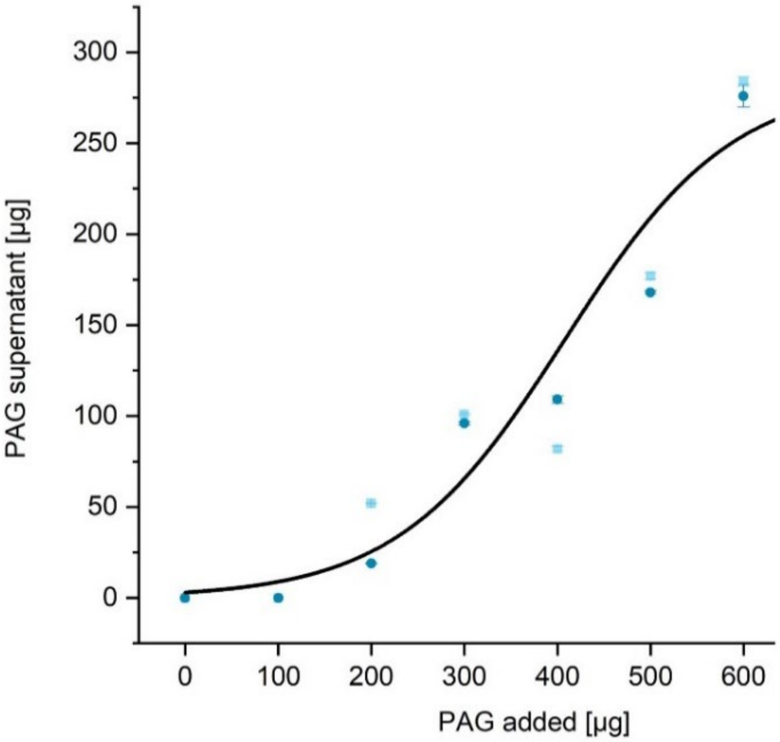

Figure S4. Determination of the protein binding capacity (binding buffer TBS40 dark blue, TBS20 light blue). A maximum of around 300 mg His6-PAG in the supernatant was determined when incubating 100 mg functionalized corundum with 600 µg His6-PAG.

5. Ni-NTA Agarose Protocol Jena BioScience (AC-501-10)

50 µL of agarose beads in 0,5 mL native lysis buffer (50 mM NaH_2PO_4, 300 mM NaCl, 10 mM imidazole, pH 8.0) were equilibrated and washed. The supernatant was discarded after a centrifugation step (6000 rpm, 5 min). Then 1 mL cytoplasm containing 1 mg spiked 6His-PAG and 0,5 mL native lysis buffer were incubated for 1 h at room temperature. The supernatant was removed and collected for the SDS-PAGE. The particles were washed 3 times with 0.5 mL washing buffer (50 mM NaH_2PO_4, 300 mM NaCl, 20 mM imidazole, pH 8.0). Elution took place for 10 min with 50 µL elution buffer (50 mM NaH_2PO_4, 300 mM NaCl, 250 mM imidazole pH 8.0). Particles were centrifuged and the eluate was taken for SDS-PAGE analysis.

Source: https://www.jenabioscience.com/files/jenabioscience/datasheet_extern/AC-501.pdf (Accessed 03.04.2023)

6. Production of His-6-tagged MBP-mSA2 and blanks in different E. coli strains

BL21(DE3) pLysS and NiCo21(DE3) competent *E. coli* cells were transformed with vector pET-MBP-mSA2 and selected on LB plates supplemented with 30 µg/mL kanamycin and 2 % glucose. Inoculated liquid cultures were grown in 4 mL of LB medium containing already mentioned additives overnight shaking at 200 rpm and 37 °C. Prewarmed TB medium (12 g/L tryptone, 24 g/L yeast extract, 4 mL/L glycerol, 17 mM potassium hydrogen phosphate, 72 mM dipotassium hydrogen phosphate) was inoculated 1:100 with overnight culture and grown under stated conditions until the optical density at 600 nm (OD_{600}) reached 0.6. Protein expression was induced with 1 mM isopropyl β-D-1-

thiogalactopyranoside (IPTG, final conc.) and allowed to proceed overnight shaking at 200 rpm and 16 °C. The bacterial cells were harvested by centrifugation (10,000 x g, 4 °C, 20 min) and frozen at -20 °C. For cell disruption, the pellet was resuspended in lysis buffer (300 mM NaCl, 46.6 mM Na_2HPO_4, 1.9 mM NaH_2PO_4, 10 mM imidazole, pH 8) with additional 1 mg/mL lysozyme as well as protease inhibitors and lysed via sonication. Insoluble cell debris was collected through centrifugation (10,000 x g, 4 °C, 20 min) and subsequently, a soluble fraction of the cell lysate was taken off and filtered through a 0.22 µm filter before recombinant protein purification.

For the production of blanks, single colonies of both competent cell lines were generated by dilution-plating on LB plates with 2% glucose. The further procedure was analogous to the expression conditions of pET-MBP-mSA2 without adding kanamycin during cultivation. In 1 mL blanks, 1 mg of His6-PAG was spiked and then diluted with 1 mL of TBS binding buffer containing different amounts of imidazole.

7. Production of SARS-CoV-2-S-RBD-His8 in human Expi293F cell line

Expi293F-cells (Thermo Fisher Cat#A14527) were cultivated in serum-free and ready-to-use Expi293 expression media (Thermo Fisher Cat#A1435101). Cell growth occurred in sterile, ventilated 125 mL Erlenmeyer flasks (Fisher Scientific Cat#BBV12-5) shaking at 37°C, 125 rpm with 80% humidity and 8% CO_2. The transient transfection of the cells took place with the plasmid pcDNA3-SARS-CoV-2-S-RBD-8his (Addgene #145145) [21,22] and the Gibco™ ExpiFectamine™ 293 Transfections Kit (Thermo Fisher Cat#A14524) according to the manufacturer. The cell density was adjusted to 3 x 10^6 cells/mL in a final volume of 25 mL Expi293-expression media in 125 mL Erlenmeyer flasks. For the transfection, 25 µg plasmid and 80 µL of ExpiFectamine293 were diluted in 1.5 mL Opti-MEM-Medium. Later the reagents of both tubes were homogenized, incubated for 15 min at room temperature, and added to the cell suspension dropwise. After 20 hours, 150 µL of ExpiFectamine™ 293 Transfection Enhancer 1 and 1,5 mL of ExpiFectamine™ 293 Transfection Enhancer 2 were added to the cell suspension and incubated further whilst shaking. Cells were counted each day after transfection and checked on their viability (%) (automatic cell counter EVE, VWR # 734-2675). Protein harvest was performed 4 days after transfection; cells were centrifuged for 30 min at 4000 x g and the decanted supernatant was stored at 4°C until protein purification.
